# The circ_0032822 Promotes the Proliferation of Head and Neck Squamous Cell Carcinoma Cells Through miR-141/EF3 Signaling Axis

**DOI:** 10.3389/fonc.2021.662496

**Published:** 2021-04-23

**Authors:** Shuajia Zhang, Jiahui Han, Jing Fu

**Affiliations:** ^1^ Department of Otolaryngology Head and Neck Surgery, The First People’s Hospital of Lianyungang, The Affiliated Lianyungang Hospital of Xuzhou Medical University, Lianyungang, China; ^2^ Department of Respiratory Medicine, The First People’s Hospital of Lianyungang, The Affiliated Lianyungang Hospital of Xuzhou Medical University, Lianyungang, China

**Keywords:** HNSCC, ceRNA, circRNA, cell cycle, apoptosis

## Abstract

Head and neck squamous cell carcinoma (HNSCC) refers to an epithelial malignant tumor that originates in the head and neck, and over 600,000 new cases are reported every year, However, the overall prognosis is still poor due to local recurrence and distant metastasis after surgery. The circ_0032822 has been reported upregulated in human oral squamous cell carcinoma; however, the detailed function or mechanism remains unknown. In this study, we confirmed the upregulation of circ_0032822 in HNSCC tumor tissues. Functionally, the overexpression of circ_0032822 significantly promoted the proliferation of HNSCC cell lines along with the S phase arrest and reduced apoptosis, while downregulation of circ_0032822 has the opposite effect *in vitro*. Mechanistic analysis showed that circ_0032822 acted as a competing endogenous RNA of miR-141 to diminish the repressive effect of miR-141 on its target E2F3. In conclusion, we demonstrated that circ_0032822 functions as a tumor oncogene in HNSCC and that its function is regulated *via* the miR-141/E2F3 axis.

## Introduction

Head and neck squamous cell carcinoma (HNSCC) refers to an epithelial malignant tumor that originates in the head and neck and over 600,000 new cases are reported every year (1, 2). Although advances in technology and supportive treatment have improved the quality of life of HNSCC patients, the overall prognosis is still poor due to local recurrence and distant metastasis after surgery (3, 4). Therefore, there is an urgent need to better understand the molecular mechanism of HNSCC progression to improve the prevention, diagnosis, and personalized treatment of patients.

Circular RNAs (circRNAs) are a new class of non-coding RNAs that form covalently closed continuous loops caps and tails (5, 6). Because of the closed-loop structure, circRNAs are not easily degraded by the exonuclease RNase R and show greater stability than linear RNAs (7). Based on these features, circRNAs are defined as abundant, stable, and conserved molecules and usually exhibit tissue or developmental stage-specific expression (8). CircRNA was found to participate in multiple human diseases through different approaches. It was observed that circRNAs possess protein translation ability and could also be regarded as efficient miRNA sponges, as they contain conserved miRNA target sites (9). As regulatory factors, miRNAs regulate physiological and pathological processes by blocking protein translation or inducing mRNA degradation to inhibit target gene expression and are widely involved in many biological processes, such as cell metabolism, proliferation, differentiation and apoptosis (8, 9).

Previously reported has identified that expression profile of circular RNAs in oral squamous cell carcinoma (OSCC), which was the commonest cancer in the oral maxillofacial region also was a common tumor of head and neck and is often accompanied by regional lymph node metastasis and even distant metastasis (10). Based on the CircRNA sequencing data, researchers found several dysregulated circRNA in OSCC tumor tissues, one of the most significant upregulated circRNA was circ_0032822. However, little of the function or mechanism of circ_0032822 was identified in human disease and in HNSCC. Here in the present study, we first validated the upregulation of circ_0032822 in HNSCC tumor tissues, following bioinformatics prediction analysis implied that circ_0032822 acts as the sponge of miR-141 in HNSCC cells and that it might promote metastasis through an miR-141-dependent mechanism. The subsequent experiments also suggested that E2F3 is the target of miR-141. Our findings indicated that circRNAs might exert regulatory functions in HNSCC.

## Material and Methods

### Patient Samples

Samples, including tumor tissues and corresponding adjacent tumor tissues, were obtained from patients with HNSCC from 2006 to 2019. Patients with HNSCC were diagnosed based on surgical and pathological findings in The First People’s Hospital of Lianyungang. After sample collection, liquid nitrogen was frozen and transported, and stored in a -70°C; deep cryogenic refrigerator. Written consent was obtained from the patients enrolled in this study. The clinical-stage was based on the 8th edition of the International Union Against Cancer (UICC) on the Tumor-Node-Metastasis (TNM) staging system. All experiments were performed in compliance with government policies and the Helsinki Declaration. All subjects were informed about the study and provided their consent prior to specimen collection. All experiments were approved by the ethics committee of the First People’s Hospital of Lianyungang.

### Cell Lines and Cell Culture

HNSCC cell lines, including HN-4, HN-9, HN-30, SCC-4, SCC-9, and SCC-25 cells, were purchased from the Cell Bank of Type Culture Collection of the Chinese Academy of Sciences (Shanghai, China), and were cultured and stored according to the guidelines of the cell bank. The culture medium used was Dulbecco’s modified Eagle’s medium (DMEM; Winsent, Quebec, Canada) containing 10% fetal bovine serum (FBS), 100 U/ml penicillin, and 100 µg/ml streptomycin. All the cell lines were incubated in a 5% CO_2_ humidified incubator at 37°C.

### RNA Isolation and Real-Time Quantitative PCR

TRIzol reagent was used to isolate RNA from colorectal tissues or cell lines according to the manufacturer’s protocol. Next, 1 μg RNA was used to synthesize cDNA, followed by gene expression analysis on the ABI 7900 qPCR system. Relative expression was normalized to that of GAPDH. The primers used for GAPDH and circ_0032822 were as follows: GAPDH: forward: 5ʹ-GGAGCGAGATCCCTCCAAAAT-3ʹ, reverse: 5ʹ-GGCTGTTGTCATACTTCTCATGG-3ʹ; circ_0032822: forward: 5’-AGAAAGGCAGGAGCAGCTT-3’, reverse: 5’-TCCAGCTGACCACGATGA-3’.

### Cell Proliferation Assay

Cells transfected for 24 h with miRNA mimic or stably transduced cells were seeded into 96-well plates at a density of 1000 cells per well in triplicate. The cells were harvested, and 10 μl of CCK-8 reagent (Dojindo, Kumamoto, Japan) was added to 100 μl of culture medium. The cells were subsequently incubated for 2 h at 37°C, and the optical density was measured at 450 nm using a microplate reader (SpectraMax i3, Molecular Devices, USA).

For colony formation assay, treated cells were seeded onto 6-well plates (500 cells per well). After 2 weeks, cells were fixed with 4% paraformaldehyde for 30 minutes and finally stained with crystal violet. Colonies were counted by the naked eye after rinsed twice by phosphate buffer saline (PBS).

### Flow Cytometry Analysis of Cell Cycle and Apoptosis

For cell-cycle analysis, cells were subjected to serum starvation for cell cycle synchronization. The cells at the logarithmic growth period were harvested and fixed in 70% ethanol overnight at −20°C. The cells were washed and incubated in propidium iodide (PI) (Multi-Science) and analyzed by flow cytometry. Apoptosis was analyzed by using the Annexin V–APC/7-AAD apoptosis kit and flow cytometry, according to the manufacturer’s instructions.

### Mitochondrial Membrane Potential Detection

The Mitochondrial membrane potential detection was conducted by using Mitochondrial Membrane Potential and Apoptosis Detection Kit with Mito-Tracker Red CMXRos and Annexin V-FITC according to the manufacturer’s instructions (MKBio, Shanghai, China). In brief, after washing adherent cells with PBS, Annexin V-FITC and Mito-Tracker Red CMXRos staining were added, respectively. Hoechst 33342 was added after 30 minutes of dark incubation for nucleus staining. Fluorescence intensity was observed under a fluorescence microscope.

### *In Vivo* Experiments

Six-week-old male nude mice (NOD/SCID background) were randomly divided into indicated groups (n=6) before inoculation. A total of 5×10^6^ cells transfected with indicated vectors were subcutaneously into the left or the right flanks of mice. Tumors were allowed to grow for 5 weeks. All mice were sacrificed and tumor volume was calculated (length × width^2^ × 0.5). All the *in vivo* experiments were repeated three times. All animal experiments were conducted in accordance with the Institutional Animal Care and Use Committee guidelines at The First People’s Hospital of Lianyungang.

### RNA Immunoprecipitation Assay

RNA immunoprecipitation (RIP) assay was performed according to a previously reported protocol. The Magna RIP RNA-Binding Protein Immunoprecipitation Kit (Millipore, CA, USA) was used, and the assay was conducted according to the manufacturer’s instructions. As the negative control, the IgG antibody was incubated in the suspension of magnetic beads with rotation for 30 min. After washing with ice-cold RIP wash buffer, the immunoprecipitated RNA was purified and detected by qRT-PCR.

### Bioinformatics Analysis

The sequence of circ_0032822 was obtained from circbase (http://www.circbase.org), and TargetScan (http://www.targetscan.org/), and miRanda (http://www.microrna.org/) were used to predict the binding sites between circ_0032822 and miRNAs, and the potential target mRNAs of miR-141.

### Dual-Luciferase Reporter Assay

The full-length sequence of circ_0032822 predicted to interact with miRNA or the mutated sequence with the predicted target sites was inserted into the *Hin*dIII and *Sac*I sites of the pMIR-REPORT luciferase vector (GenScript, Nanjing, China). The cell lines cultured in 24−well plates were co-transfected with pMIR-REPORT vectors containing either the wild-type or mutated segments along with the control vector, and the pRL-TK vector containing Renilla luciferase were used for normalization. The cells were co−transfected with the precursor microRNA mimics and the control group. Assays were performed to determine the gene expression level.

### Western Blotting Assay

Proteins were extracted from cells and tissues according to the manufacturer’s protocol (KeyGEN BioTech, Jiangsu, China). In brief, after extraction with RIPA buffer with protease inhibitor and phosphatase inhibitor cocktails (Pierce Biotechnology, Rockford, IL, USA) and quantification with a BCA kit (Thermo Fisher Scientific, Waltham, MA, USA), proteins from cell lysates or tissue lysates were equally loaded onto each well of SDS-PAGE. After electrophoresis, the proteins were transferred onto a membrane, blocked with 5% non-fat milk in PBST for 1 h, and then incubated with diluted primary antibodies at 4°C overnight. The protein expression levels were detected by ECL Plus (Millipore, Billerica, MA, USA) with a bio-imaging system.

### Statistical Analysis

All statistical analyses were performed using SPSS 13.0 software (Chicago, IL, USA) and GraphPad Prism software (La Jolla, CA, USA). The chi-square test was used to analyze the correlation between circRNA expression levels and clinical features of the patients. The differences between groups were analyzed using an unpaired t-test when only two groups were compared and a one-way ANOVA analysis of variance when more than two groups were compared. Survival curves were calculated using the Kaplan-Meier method, and the significance was determined by the log-rank test. Pearson’s correlation analysis was performed to determine the correlation between the expression of circRNA, miRNA, and mRNA. Differences were considered to be statistically significant at P < 0.05.

## Results

### Increased circ_0032822 in HNSCC Tumor Tissues

**Figure 1:**
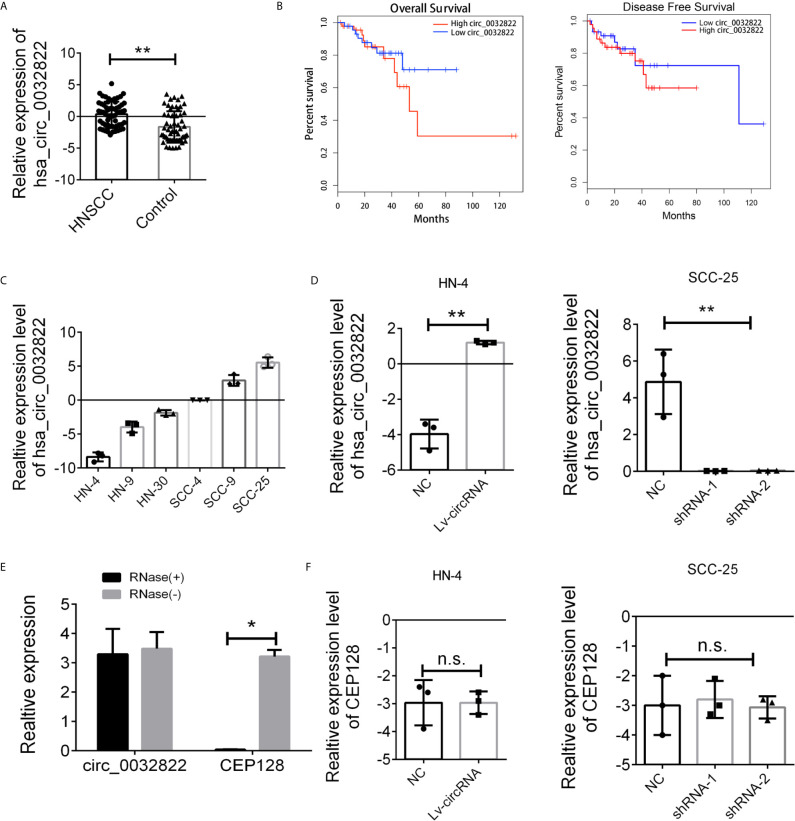
Expression level of circ_0032822 in HNSCC tissues and cell lines. **(A)** The expression of circ_0032822 in HNSCC tissues (n=60) were detected by RT-PCR. **(B)** The overall survival and recurrence-free survival rates of HNSCC patients were compared between higher circ_0032822 and lower circ_0032822 groups. **(C)** The expression of circ_0032822 in HNSCC cell lines. **(D)** Ovexpression and knocking-down efficiency in cell lines. **(E)** The PCR analysis confirmed that circ_0032822 resisted to RNase R digestion. **(F)** Expression of linear form of circ_0032822. Data were presented as plot of the mean with SEM, * indicated p < 0.05, ** indicated P < 0.01, n.s., no significant.

As circ_0032822 was found to be upregulated in OSCC tissues, we firstly investigated the expression of circ_0032822 in HNSCC tumor tissues and the adjacent corresponding tumor tissues. As shown in **Figure 1A**, we found that circ_0032822 expression was increased in tumor tissues. Based on the clinicopathological characteristics of the enrolled patients, it was found that circ_0032822 was highly associated with tumor size instead of TNM stage or lymph node metastasis (**Table 1**). Furthermore, Kaplan-Meier and log-rank test analyses suggested a positive correlation between the tumoral circ_0032822 expression and significantly reduced overall survival (OS) and recurrence-free survival (RFS) rates (P = 0.013 for OS and P = 0.021 for RFS) (**Figure 1B**).

**Table 1:** Correlation between circRNA_0032822 expression and clinicopathological parameters in head and neck squamous cell carcinoma (HNSCC) patients.

Characteristics	circRNA_0032822 expression		*P* value^a^
	High	Low	
**N**	30	30	
**Age(year)**			0.606
≥60	14	16	
<60	16	14	
**Gender**			0.793
Male	18	17	
Female	12	13	
**Tumor site**			0.796
Oral cavity/Oropharynx	15	16	
Larynx/Hypopharynx	15	14	
**Histologic grade**			0.953
G1	13	14	
G2	9	8	
G3	8	8	
**Tumor Size (cm)**			0.002
≥3	22	10	
<3	8	20	
**TNM stage(I:II:III)**	15:11:4	14:10:6	0.786
**Lymph node metastasis**			0.795
Yes	17	16	
No	13	14	

a Chi-square test.

Next, we aimed to conduct the loss-of-function and gain-of-function assay to detect the endogenous expression of the circ_0032822 in HNSCC cell lines. HN4 was selected as the cell model for overexpression based on the lowest expression level while SCC-25 was considered for knocking down by shRNA technology (**Figure 1C**). Next, HN4 was treated with a circ_0032822-overexpressing lentivirus vector and SCC-25 was treated with two independent shRNA lentivirus vectors. The infection efficiency was measured by RT-PCR (**Figure 1D**). Since circ_0032822 was little known in human disease or cells, the RNase exonuclease was applied to text the stable expression of circRNA. We found that circ_0032822 was resistant to RNase after digestion of RNA instead of the liner form (**Figure 1E**). Also, we also confirmed the knock-down lentivirus and the overexpression lentivirus were all specifically targeted at circ_0032822, but not its linear form, CEP128 mRNA (**Figure 1F**).

### The circ_0032822 Promoted the Proliferation of HNSCC Cells *In Vitro*

**Figure 2:**
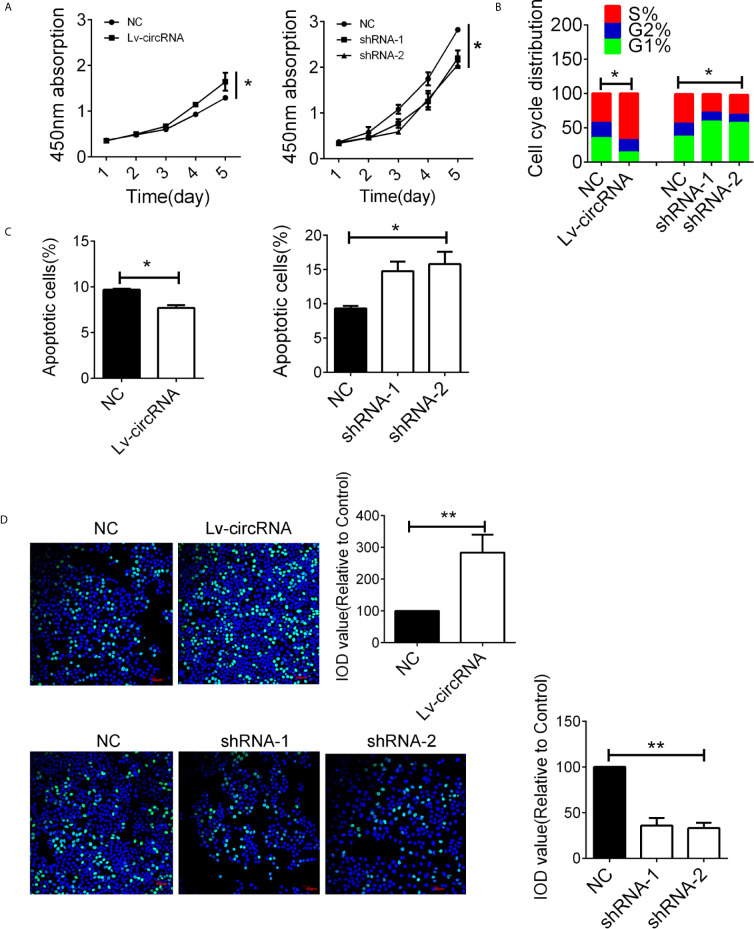
The circ_0032822 promoted cell function of HNSCC **(A)** CCK8 assay in cells treated with circ_0032822 overexpression or knocking-down lentivirus. The F value for the ANOVA analysis between shRNAs and control group was 10.2. **(B)** Cell cycle distribution in cells. The F value for the ANOVA analysis between shRNAs and control group was 12.1. **(C)** Cell apoptosis detected by flow cytometry. The F value for the ANOVA analysis between shRNAs and control group was 14.6. **(D)** EDU assay in HNSCC cells. The F value for the ANOVA analysis between shRNAs and control group was 28.1. * indicated P < 0.05, ** indicated P < 0.01.

The high expression of circ_0032822 in HNSCC tissues indicated its tumorigenic effect in HNSCC cells. In order to evaluate the circ_0032822 function in HNSCC cells, the CCK8 assay was employed. We found that circ_0032822 could promote the proliferation of HNSCC cells while knocking down of circ_0032822 induced a suppression of tumor cells **(** **Figure 2A** **).** Besides, the cell cycle was also investigated in different groups mentioned above. We also found a S phage arrest when cells were upregulated with circ_0032822; however, the G1 phage arrest was obtained when circ_0032822 was knocking down **(** **Figure 2B**; **Figure S1A** **)**. Consistently, we also found a reduced apoptotic cell when circ_0032822 was overexpressed and the apoptosis of the cell could be promoted by treating cells with circ_0032822 shRNAs **(** **Figures 2C**; **S1B** **)**. The EDU assay also confirmed the similar function of circ_0032822 in HSNCC cells **(** **Figure 2D** **)**.

**Figure 3:**
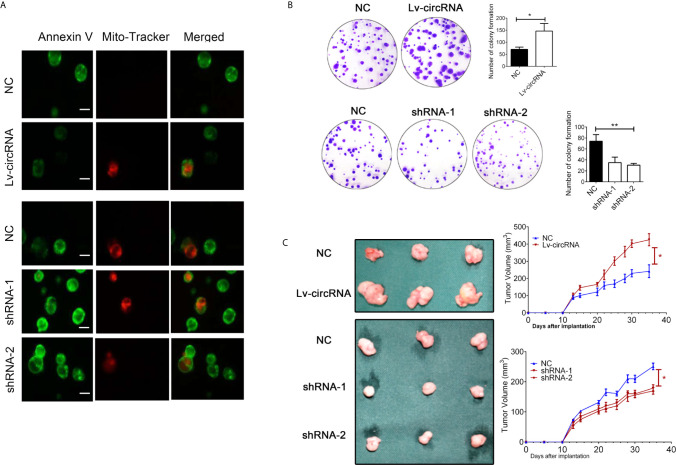
The circ_0032822 promoted cell proliferation while reduced apoptosis of HNSCC. **(A)** The mitochondrial membrane potential detection through the immunofluorescence. Annexin V-FITC (green), Mito-Tracker Red CMXRos (red). **(B)** Colony formation assay. The F value for the ANOVA analysis between shRNAs and control group was 20.1. **(C)** Tumor size and growth curve of colorectal cancer cells treated with circ_0032822 overexpression or knocking down lentivirus. *indicated P < 0.05, ** indicated P < 0.01.

Besides, we also conducted the mitochondrial membrane potential detection through immunofluorescence to confirm cell apoptosis. As presented in **Figure 3A**, we found that upregulated circ_0032822 will reduce apoptosis of colorectal cells while knocking-down of circ_0032822 will promote the apoptosis. We also performed colony forming assay to further detect cell proliferation. The number of colony formation was increased when circ_0032822 was up-regulated while could be attenuated by the suppressing of circ_0032822 **(** **Figure 3B** **)**. A subcutaneous xenograft model was constructed by subcutaneous injection of colorectal cancer cells in NOD/SCID mice. We found the tumor growth was promoted by increasing the level of circ_0032822 in colorectal cancer cells while the tumor growth was suppressed by knocking down of circ_0032822 **(** **Figure 3C** **)**.

### Identification of miRNA Candidates That Can Bind to circ_0032822

**Figure 4:**
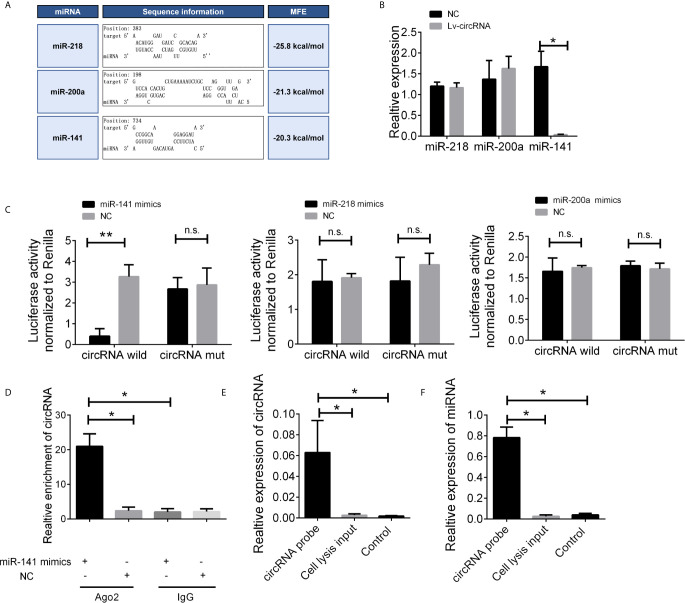
The circ_0032822 could be bound by miR-141. **(A)** Potential miRNA prediction. **(B)** Relative expression of miRNA in cells treated with circ_0032822 overexpression lentivirus. **(C)** Dual-luciferase reporter assay in cells treated with circ_0032822 and candidate miRNA mimics. **(D)** RIP analysis showed that circ_0032822 was abundantly pulled down by anti-Ago2 antibodies when transfected with miR-141 mimics, compared to the NC or IgG group. **(E, F)** Biotin-coupled probe pull down assay confirmed that miR-141 was effectively enriched by circ_0032822. Data were presented as plot of the mean with SEM. * indicated P < 0.05, ** indicated P < 0.01, n.s., no significant.

Given that circRNAs may act as competing endogenous RNAs (ceRNAs) of miRNAs to regulate mRNA expression, we next assessed the potential targets of circ_0032822 through a ceRNA-dependent mechanism. Furthermore, by using the bioinformatics tool Miranda, we predicted and screened the top three possible sponge miRNAs, including miR-218, miR-200a, and miR-141 (**Figure 4A**). The subsequent qRT-PCR analysis showed that the upregulation of circ_0032822 decreased the expression of only miR-141 but not of miR-218 or miR-200a (**Figure 4B**). Next, the dual luciferase reporter assay was conducted to determine whether circ_0032822 could directly bind to the miRNA candidate. As shown in **Figure 4C**, we found that only the luciferase intensity of miR-141 was significantly suppressed. The RIP analysis also showed that more circ_0032822 was pulled down by anti-Ago2 antibody when transfected with miR-141 mimics in DLD-1 cells as compared to the miR-141 control group and the IgG group (**Figure 4D**). A biotin-coupled circ_0032822 probe was also designed for the pull-down assay, and the results showed that miR-141 was effectively enriched by circ_0032822 (**Figures 4E, F** **)**.

### miR-141 Directly Targets E2F3

**Figure 5:**
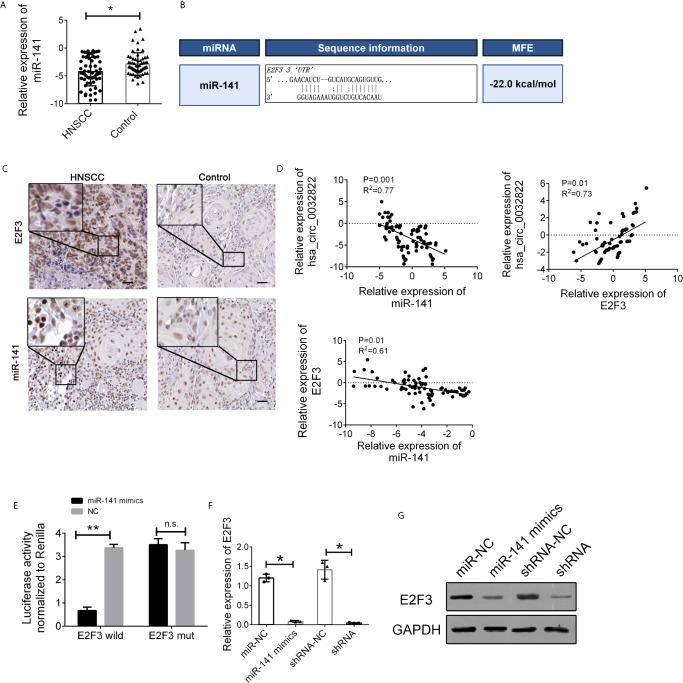
miR-141 directly targeted E2F3. **(A)** Relative expression of miR-141 in tissues samples of HNSCC. **(B)** Detailed binding site for miR-141 and E2F3. **(C)** IHC stains of miR-141 and E2F3 in HNSCC tumor tissues. **(D)** Pearson correlation analysis for circ_0032822/miR-141/E2F3 in tissues. Data were presented as plot of the mean with SD with log-transformed. **(E)** Dual-luciferase reporter assay in cells treated with E2F3 3’UTR and candidate miR-141 mimics. **(F)** mRNA expression of E2F3. **(G)** Protein expression of E2F3. Data were presented as plot of the mean with SEM. * indicated P < 0.05, ** indicated P < 0.01, n.s., no significant.

To further elucidate the regulatory relationship between circ_0032822 and miR-141, the expression levels of miR-141 in the tissue’s samples of HNSCC patients were also determined. We found that miR-141 expression was downregulated in tumor tissues of HNSCC patients (**Figure 5A**). MiR-141 was reported to inhibit gastric cancer proliferation by interacting with MEG3 and suppressing E2F3 expression (11). Bioinformatics analysis also revealed that E2F3 was the direct target of miR-141 with the highest binding ability (**Figure 5B**). We also confirmed the expression of miR-141 and E2F3 in the histological images of HNSCC. We found that miR-141 was down-regulated in tumor tissues while E2F3 was up-regulated (**Figure 5C**). Therefore, we speculated that circ_0032822 may regulate the metastatic process through the miR-141/E2F3 pathway. Pearson’s correlation analysis was also used to confirm this finding. The results showed an inverse correlation between circ_0032822 and miR-141, a positive correlation between circ_0032822 and E2F3, and an inverse correlation between miR-141 and E2F3 (**Figure 5D**). A subsequent dual-luciferase reporter assay indicated that miR-141 could directly bind to E2F3 (**Figure 5E**). We also investigated the expression level of E2F3 with miR-141 mimics transfection and circ_0032822 shRNA, we found that both the mRNA and protein expression of E2F3 was suppressed either treating cells with miR-141 mimics transfection or circ_0032822 shRNA (**Figures 5F, G** **)**.

**Figure 6:**
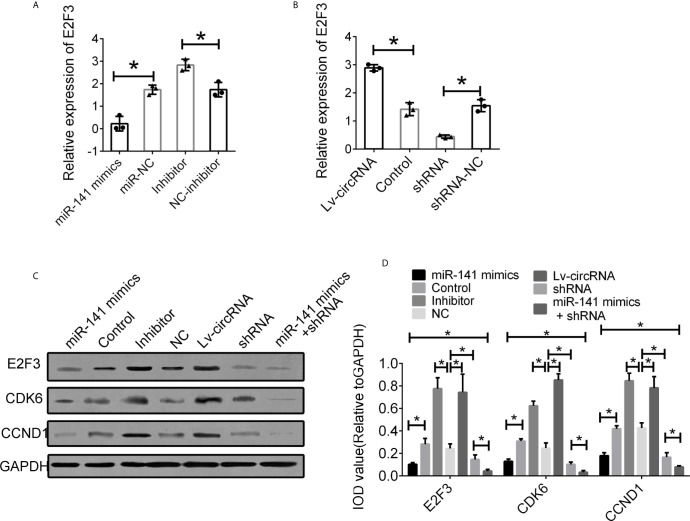
Interaction between circ_0032822, miR-141 and E2F3. **(A)** Relative mRNA expression of E2F3 in cells treated with miR-141 mimics or inhibitor. **(B)** Relative mRNA expression of E2F3 in cells treated with circ_0032822 overexpression or shRNA lentivirus. **(C)** Protein level of E2F3 in cells with different treatment. **(D)** Integral optical density analysis of immunoblots. The F value for the ANOVA analysis between shRNAs and control group was 13.2, 12.7, 11.6, respectively. Data were presented as mean with SEM. * indicated P < 0.05.

We further investigated the interaction between circ_0032822, miR-141, and E2F3. First, we detected the expression of E2F3 in cells treated with miR-141 mimics and an miR-141 inhibitor. The expression of E2F3 was suppressed following miR-141 overexpression, while E2F3 expression was upregulated through the inhibition of miR-141 **(** **Figure 6A** **)**. Furthermore, the increased level of circ_0032822 also promoted the expression of E2F3, while the suppression of circ_0032822 expression downregulated E2F3 expression **(** **Figure 6B** **)**. The protein level of E2F3 and the downstream signalling pathway was also confirmed to be consistent with its mRNA level mentioned above. We also co-transfected the cells with miR-141 mimics and circ_0032822 shRNA. the expression of E2F3 and the downstream signalling pathway presented stronger inhibition **(** **Figures 6C, D** **)**.

## Discussion

Head and neck squamous cell carcinoma (HNSCC) is the sixth most common type of human malignancy and involves carcinoma of several anatomic sites, such as lip, oral cavity, pharynx (nasopharynx, oropharynx, hypopharynx), and larynx, with an annual incidence of ~600,000 per year (12, 13). Although the diagnosis and treatment have advanced in recent years, HNSCC still has a high incidence and mortality rate in developing countries (14, 15). Therefore, identifying new molecular targets is a key step in improving the diagnosis, prognosis, and treatment of HNSCC.

CircRNA is considered a novel biomarker and therapeutic target for human cancer. Numerous studies have shown that circRNAs have an important role in human cancers (16, 17). CircRNAs function as a miRNA sponge; it may interact with proteins and regulate protein translation and gene transcription. Researchers have developed a database to predict the potential binding of circRNA by RNA binding protein. For example, the Circular RNA Interactome database designed by NIH (https://circinteractome.nia.nih.gov/) provided 109 datasets of RNA-binding proteins (RBPs) and queried circRNAs for RNA-binding sites. This computational tool enables the prediction and mapping of binding sites for RBPs and miRNAs on reported circRNAs (18). Increasing evidence has shown that circRNAs play a crucial role in carcinogenesis, cancer progression, and clinical outcomes of various human cancers (19). For example, researchers found Circular RNA 103862 promoted proliferation and invasion of laryngeal squamous cell carcinoma cells through the miR-493-5p/GOLM1 axis (12). Besides, CircRNA_ACAP2 could suppress EMT in head and neck squamous cell carcinoma by targeting the miR-21-5p/STAT3 signalling axis (20). Based on the important function of circRNA in HNSCC, The CircRNA sequencing was performed in 6 pairs of samples of OSCC and normal oral mucosal tissues. Among the 122 dysregulated circRNAs, 10 circRNAs were identified for the first time as novel circRNAs. Expression of hsa_circ_0000857, hsa_circ_0001470, hsa_circ_0001766, hsa_circ_0004390, hsa_circ_0005050, hsa_circ_0006151, hsa_circ_0008603, hsa_circ_0032822, hsa_circ_0104368, hsa_circSENP2 were identified with different expression in OSCC tissues and normal oral mucosal tissues. From these results, we detailed analyzed that hsa_circ_0001766 has been deeply investigated while circ_0032822 presented a significant difference and with little known in OSCC or HNSCC.

As a member of the E2F family, E2F3 was involved in cell proliferation and regulation. Moreover, E2F3 could form a dimer with cyclin D1 and participate in the regulation of the cell cycle, which is related to a variety of oncogenic and tumor suppressor genes. It has been reported that miR-34a can regulate tumor angiogenesis by directly inhibiting angiogenic functions of endothelial cells by downregulating several key proteins including E2F3, SIRT1 and CDK4 in HNSCC (21). Besides, a suggestive relationship between E2F3 and survival indicated that a greater expression difference of E2F3 between normal and tumor tissue will possibly predict shorter survival of the patient affected by HNSCC (22). The above pieces of evidence were consistent with our results; the suppression of E2F3 was caused by overexpression of miR-141 and circ_0032822 and was accompanied by the attenuate of cell proliferation and cell cycle rearrangement. Besides, CDK6 and CCND1 were the downstream of E2F3, and were the most widely studied indicators of cell proliferation, which is involved in the occurrence and development of tumors (23). The expression of CDK6 and CCND1 was altered along with the regulation of E2F3.

In conclusion, in the present study, we outlined the function of circ_0032822 during the development of HNSCC. The circ_0032822 was identified as a tumor-promoting noncoding RNA in HNSCC through the miR-141/E2F3-dependent mechanism. These findings suggest that circ_0032822 could be used as a potential therapeutic target for HNSCC. Although this study confirmed the carcinogenic effect of circ_0032822 on HNSCC, it was not possible to exclude that other circRNAs were involved in the carcinogenesis and progression of HNSCC. Follow-up studies should add the RNA-seq or proteomics studies in HNSCC cells with circ_0032822 downregulation and overexpression to reveal the deeper mechanisms of circ_0032822 *in vivo*.

## Data Availability Statement

The raw data supporting the conclusions of this article will be made available by the authors, without undue reservation.

## Ethics Statement

The studies involving human participants were reviewed and approved by The Ethics Committee of the First People’s Hospital of Lianyungang. The patients/participants provided their written informed consent to participate in this study. The animal study was reviewed and approved by All animal experiments were conducted in accordance with the Institutional Animal Care and Use Committee guidelines at The First People’s Hospital of Lianyungang.

## Author Contributions

Conceived and designed the experiments: SZ, JH, and JF. Performed the experiments: SZ. Analyzed the data: JH. Contributed reagents/materials/analysis tools: SZ and JH. All authors contributed to the article and approved the submitted version.

## Funding

This work was supported in part by the Foundation of the First People’s Hospital of Lianyungang.

## Conflict of Interest

The authors declare that the research was conducted in the absence of any commercial or financial relationships that could be construed as a potential conflict of interest.
