# Supplementary material for: The circ_0032822 Promotes the Proliferation of Head and Neck Squamous Cell Carcinoma Cells Through miR-141/EF3 Signaling Axis
Source: Front Oncol. 2021 Apr 23;11:662496. doi: 10.3389/fonc.2021.662496 (PMC8107724; doi:10.3389/fonc.2021.662496)
Supplement: Supplementary file 1 [file DataSheet_1.pdf]

Supplementary Figure legend

Figure S1. Representative figures for cell cycle and apoptosis. A: Cell cycle was detected by flow cytometry. B: Cell apoptosis was detected by flow cytometry.

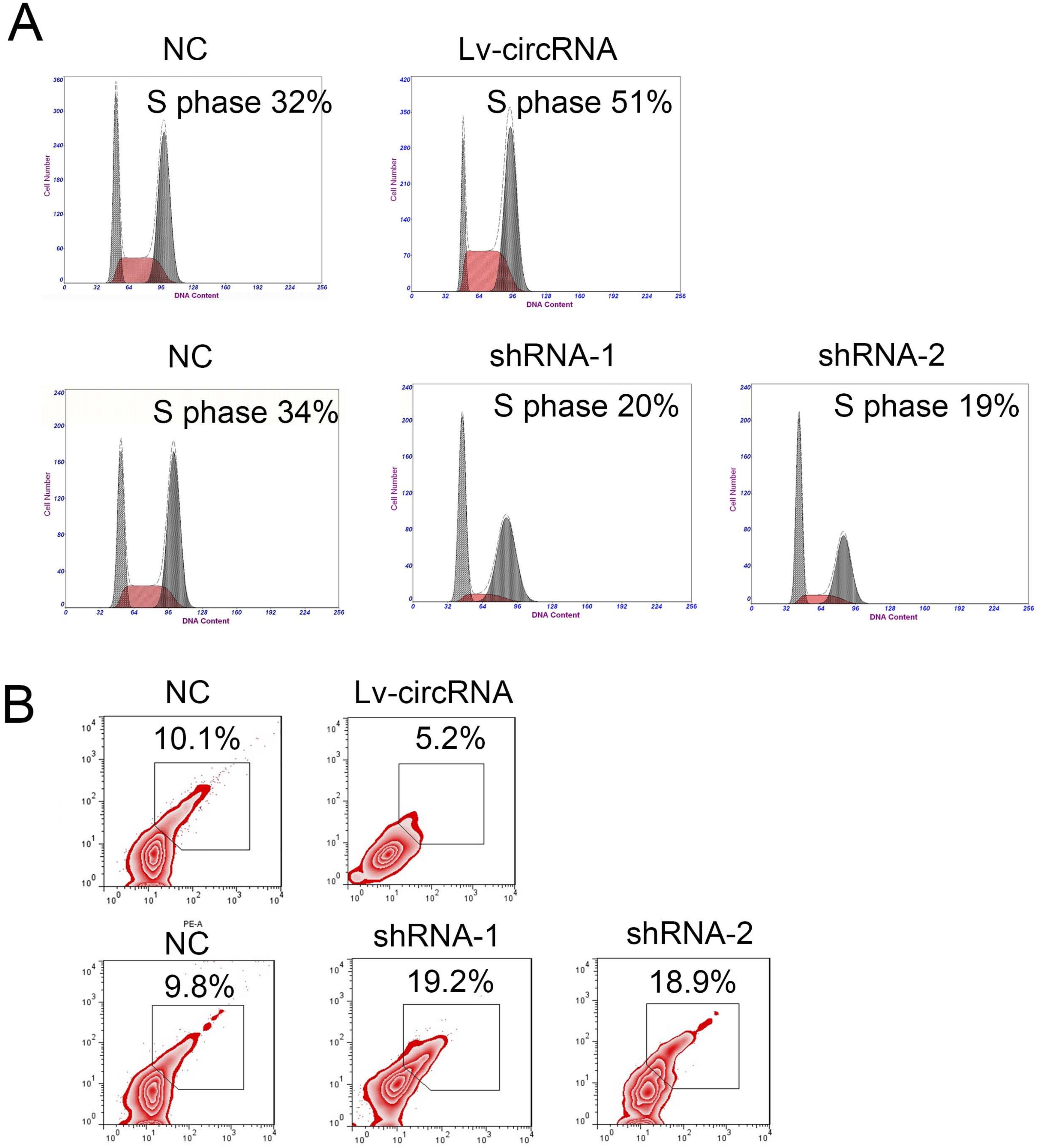
A
NC
S phase 32%
Lv-circRNA
S phase 51%
NC
S phase 34%
shRNA-1
S phase 20%
shRNA-2
S phase 19%
Cell Number
DNA Content
B
NC
10.1%
Lv-circRNA
5.2%
PE-A
NC
9.8%
shRNA-1
19.2%
shRNA-2
18.9%
